# P-1629. The Evolution of Symptom Severity and Patterns Over the First Four Years of the SARS-CoV-2 Pandemic

**DOI:** 10.1093/ofid/ofaf695.1805

**Published:** 2026-01-11

**Authors:** Maithri Reddy, Kat Schmidt, Emilie Goguet, Stephanie A Richard, John H Powers, Simon Pollett, Edward Mitre

**Affiliations:** Walter Reed Military Medical Center, Bethesda, MD; Infectious Disease Clinical Research Program, USUHS, Arlington, Virginia; HJF, USUHS, Bethesda, Maryland; Infectious Disease Clinical Research Program, Department of Preventive Medicine and Biostatistics, Uniformed Services University of the Health Sciences, Bethesda, MD, USA, Bethesda, Maryland; Support to National Institute of Allergy and Infectious Disease, Bethesda, MD; Infectious Disease Clinical Research Program, Department of Preventive Medicine and Biostatistics, Uniformed Services University of the Health Sciences, Bethesda, MD, USA, Bethesda, Maryland; Uniformed Services University, Rockville, Maryland

## Abstract

**Background:**

Understanding how symptom patterns have changed during the COVID-19 pandemic has been limited by a lack of long-term cohort data. We sought to determine if COVID-19 symptom severity, duration and patterns changed over the first four years of the pandemic.

**Figure:**
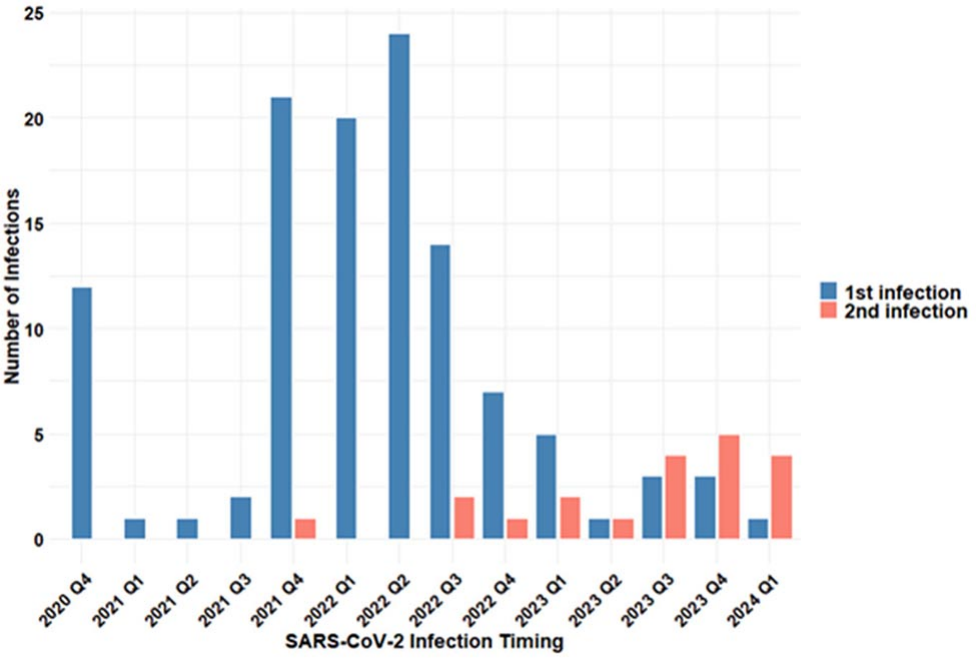
Number of SARS-CoV-2 Infections per Quarter

**Figure:**
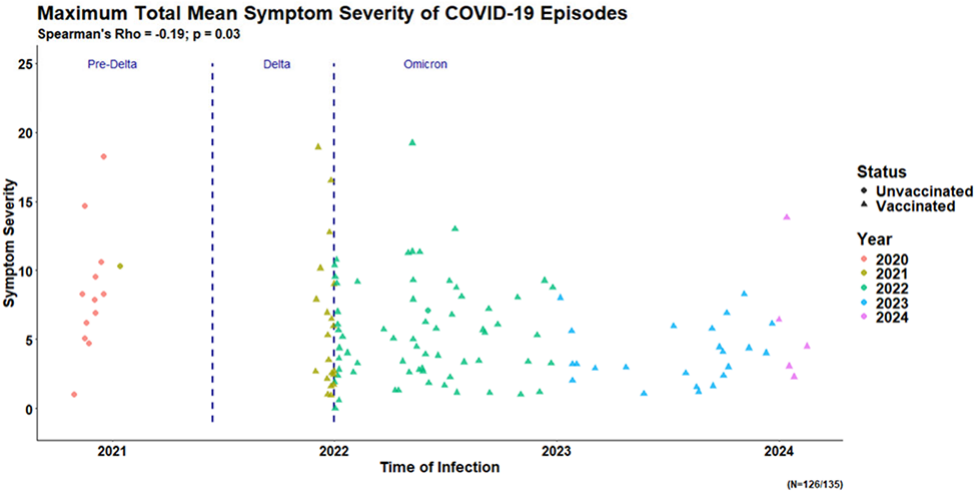

**Methods:**

A cohort of 271 generally healthy healthcare workers were enrolled from 08/2020 - 03/2021 and followed regularly until 06/2024. Participants completed a standardized and validated viral respiratory infection symptom questionnaire (FLU-PRO Plus) for confirmed COVID-19 infections. This 34-question symptom survey is grouped into 7 symptom domains: eye, nasal, throat, chest, gastrointestinal, body/systemic, and sense (smell/taste). Each domain is scored in severity from 0 to 4, and the individual domain scores summed for a total symptom severity score of 0 to 28.

**Figure:**
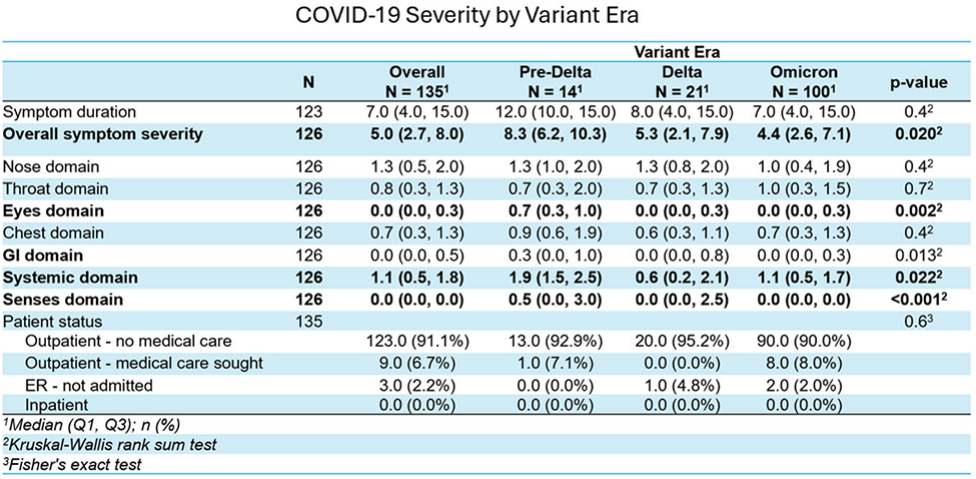

**Figure:**
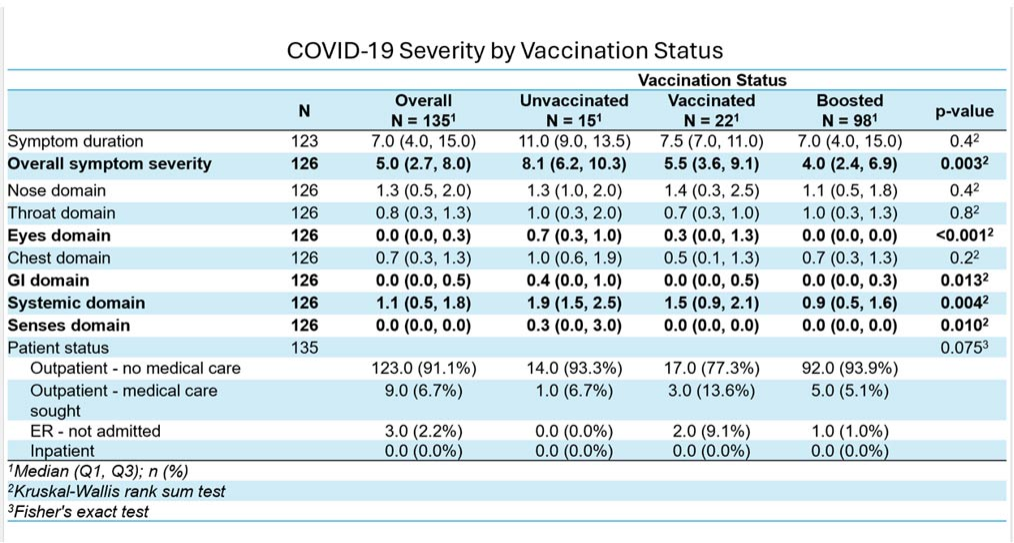

**Results:**

In our cohort, 117 participants experienced 138 confirmed SARS-CoV-2 infections. Peak symptom severity scores decreased over time from 8.3 in the pre-Delta era (2/2020 – 6/2021) to 5.3 in the Delta era (7/2021 – 12/2021) and 4.4 in the Omicron era (1/2022 – 6/2024) (p = 0.020). In contrast, there were no significant differences in total duration of symptoms with a median duration of 7 days overall. The predominant reported symptoms were related to the nose, systemic, throat, and chest domains. Over the course of the pandemic, declines were observed in symptoms that affected the sense (p < 0.001), eyes (p = 0.002), gastrointestinal (p = 0.013) and systemic (p = 0.022) domains. In bivariable analyses of vaccination status, vaccinated individuals had significantly lower total symptom severity scores (median score = 5.5) compared to unvaccinated individuals (median score = 8.1, p = 0.003). For 20 participants with repeat infections, modest but not statistically significant reductions were observed in severity and duration of symptoms during the 2^nd^ infection.

**Conclusion:**

Our results demonstrate symptom severity of SARS-CoV-2 infections has decreased over the course of the pandemic, and symptom patterns have shifted with marked reductions in eye, sense, gastrointestinal and systemic domains. These changes may be due to both acquired immunity and changes in circulating SARS-CoV-2 variants.

**Disclosures:**

All Authors: No reported disclosures

